# Supplementary material for: Engineering a synthetic pathway for maleate in Escherichia coli
Source: Nat Commun. 2017 Oct 27;8:1153. doi: 10.1038/s41467-017-01233-9 (PMC5658364; doi:10.1038/s41467-017-01233-9)
Supplement: Supplementary file 1 — Supplementary Information [file 41467_2017_1233_MOESM1_ESM.pdf]

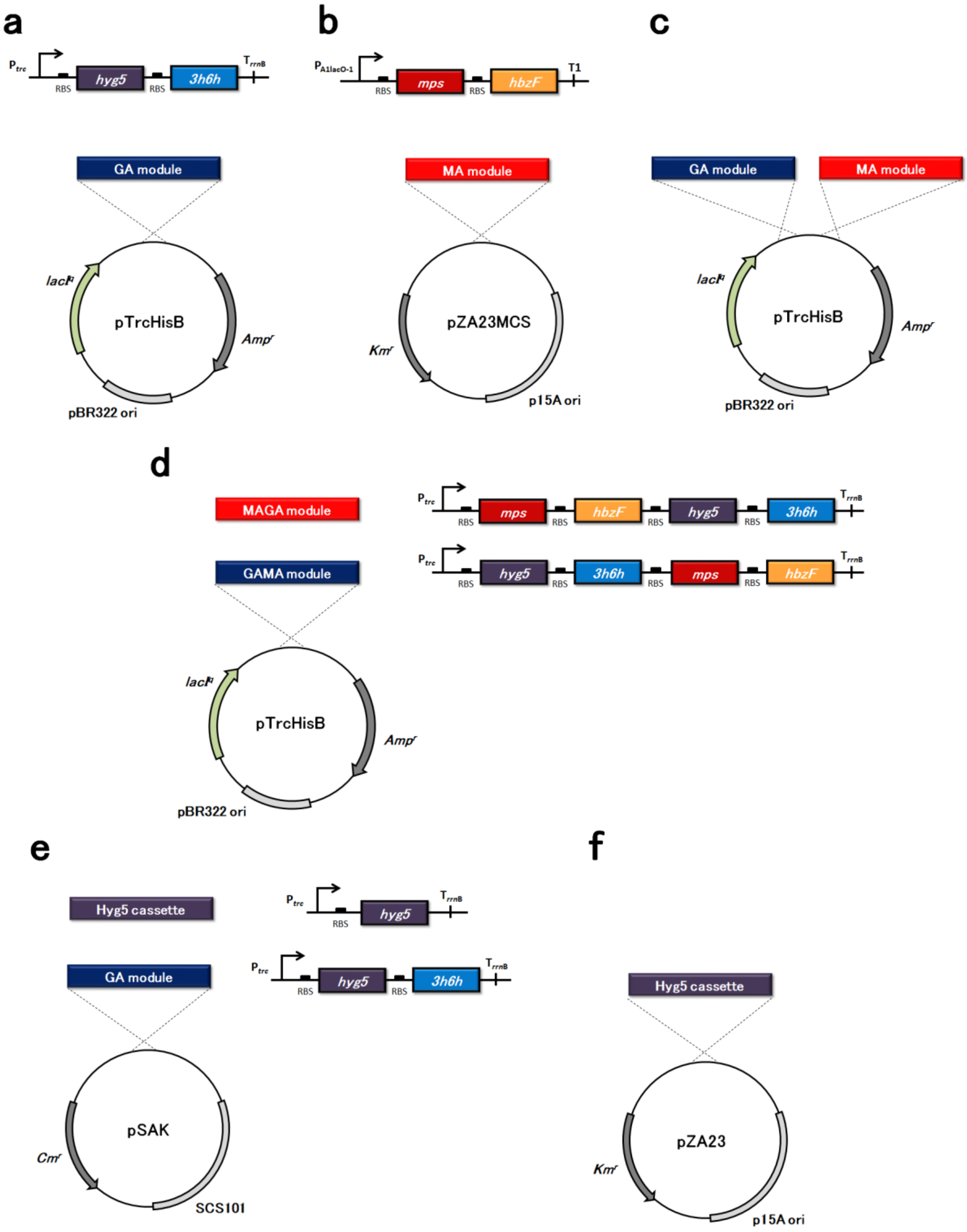
a
P trc
RBS
hyg5
RBS
3h6h
T rrnB
GA module
lacIq
pTrcHisB
Ampr
pBR322 ori
b
P A1lacO-1
RBS
mps
RBS
hbzF
T1
MA module
pZA23MCS
Kmr
p15A ori
c
GA module
MA module
lacIq
pTrcHisB
Ampr
pBR322 ori
d
MAGA module
GAMA module
mps
hbzF
hyg5
3h6h
lacIq
pTrcHisB
Ampr
pBR322 ori
e
Hyg5 cassette
GA module
pSAK
Cmr
SCS101
f
Hyg5 cassette
pZA23
Kmr
p15A ori

Supplementary Figure 1 | Maps of the plasmids constructed in this study. (**a**) pTrcHisB carrying the GA module (pTcgl3026, pT3hb6h, and pTxlnD). (**b**) pZA23MCS carrying the MA module (pZA23psal, pZA23ppu1, pZA23palc, pZA23rsp, and pZA23ppu2). (**c**) pTrcHisB carrying the GA and MA modules (pT2c101-104 and pT2c201-204). (**d**) pTrcHisB carrying the MAGA or GAMA module (pT2t01 or pT2t02). (**e**) pSAK carrying the Hyg5 cassette or GA module (pS09 or pS10). (**f**) pZA23MCS carrying the Hyg5 cassette (pA09).

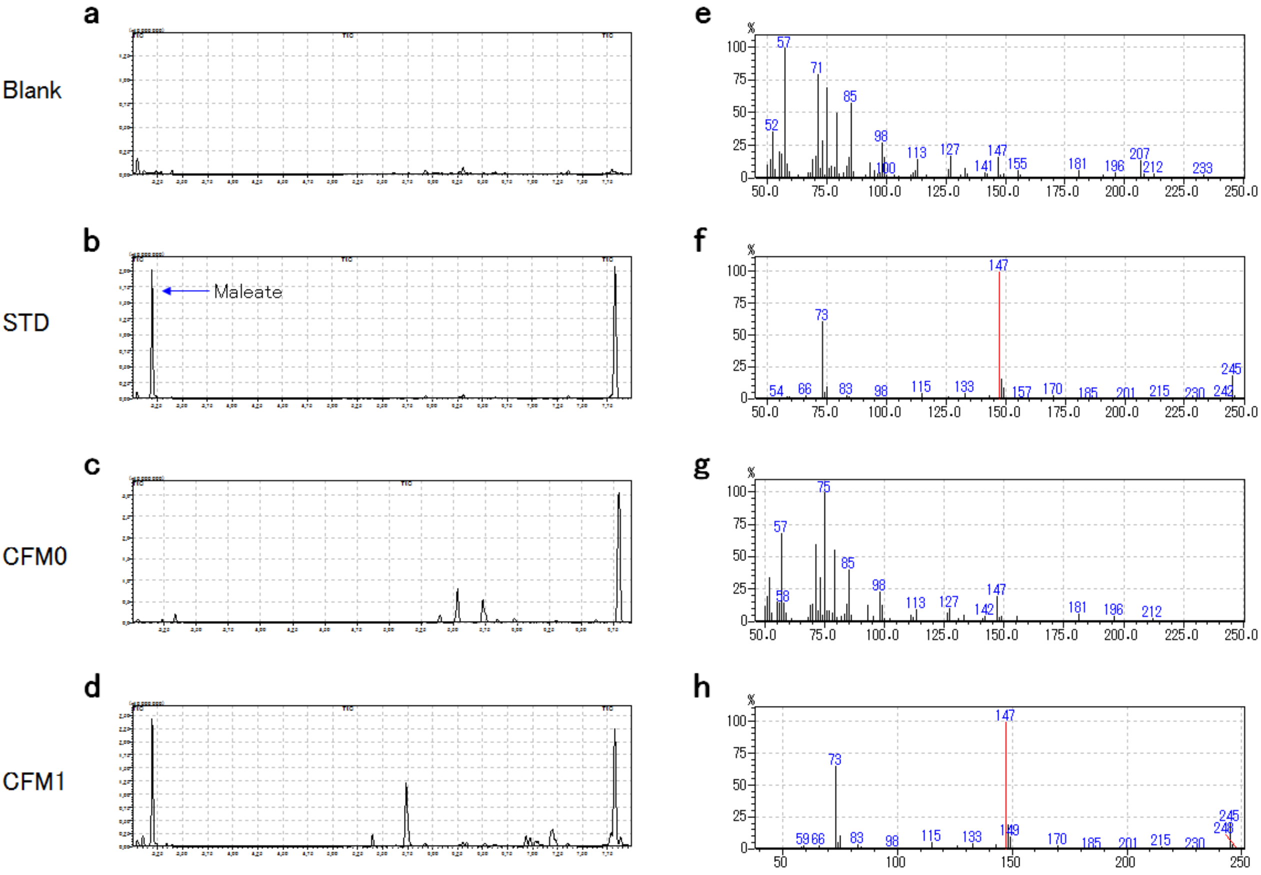

Supplementary Figure 2 | Confirmation of maleate production by gas chromatography–mass spectrometry analysis. (**a–d**) GC–MS chromatograms of maleate production. (**e–h**) MS spectra at 3.2 min in the GC–MS analysis. Each pair of **a** and **e**, **b** and **f**, **c** and **g**, and **d** and **h** presents the results for a blank, standard sample, CFM0, and CFM1, respectively.

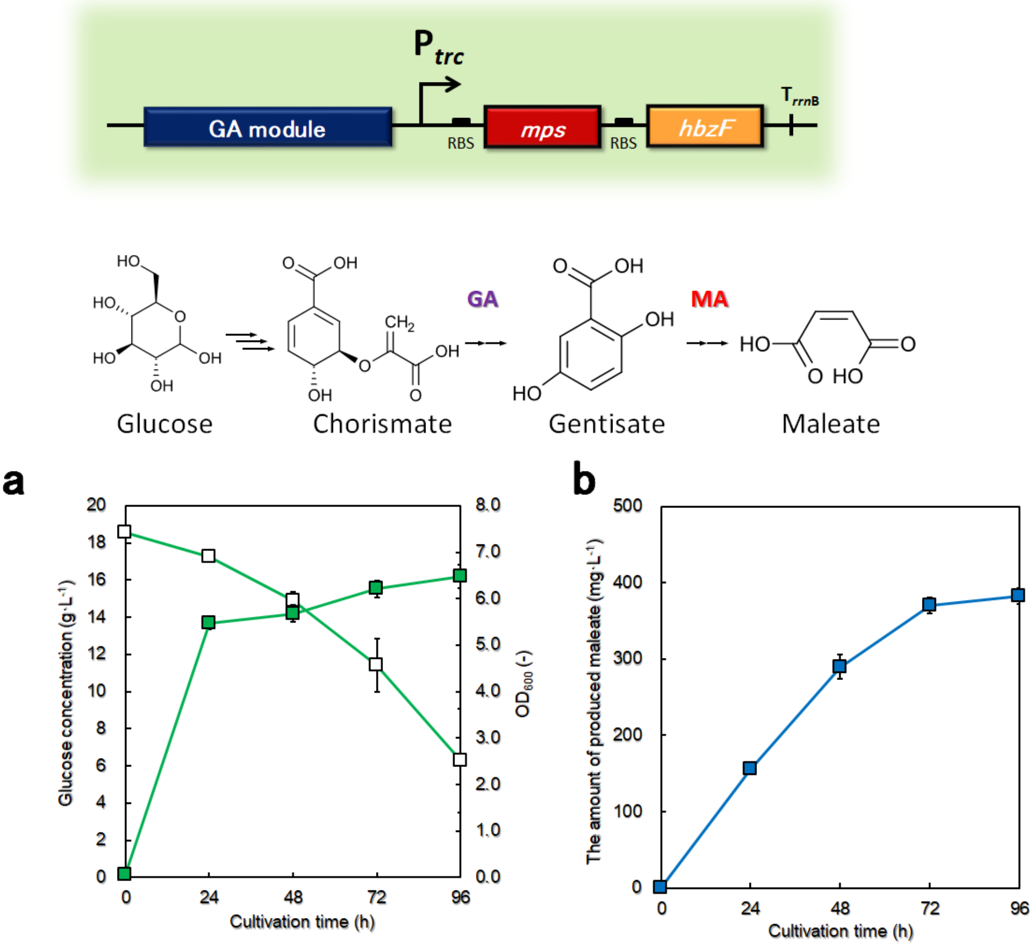

Supplementary Figure 3 | Culture profile of CFM9 during maleate production.

Time courses of (**a**) the glucose concentration (open symbols) and OD_{600} (filled symbols), and (**b**) the amount of produced maleate. Data are presented as the mean ± standard deviation of three independent experiments (n = 3).

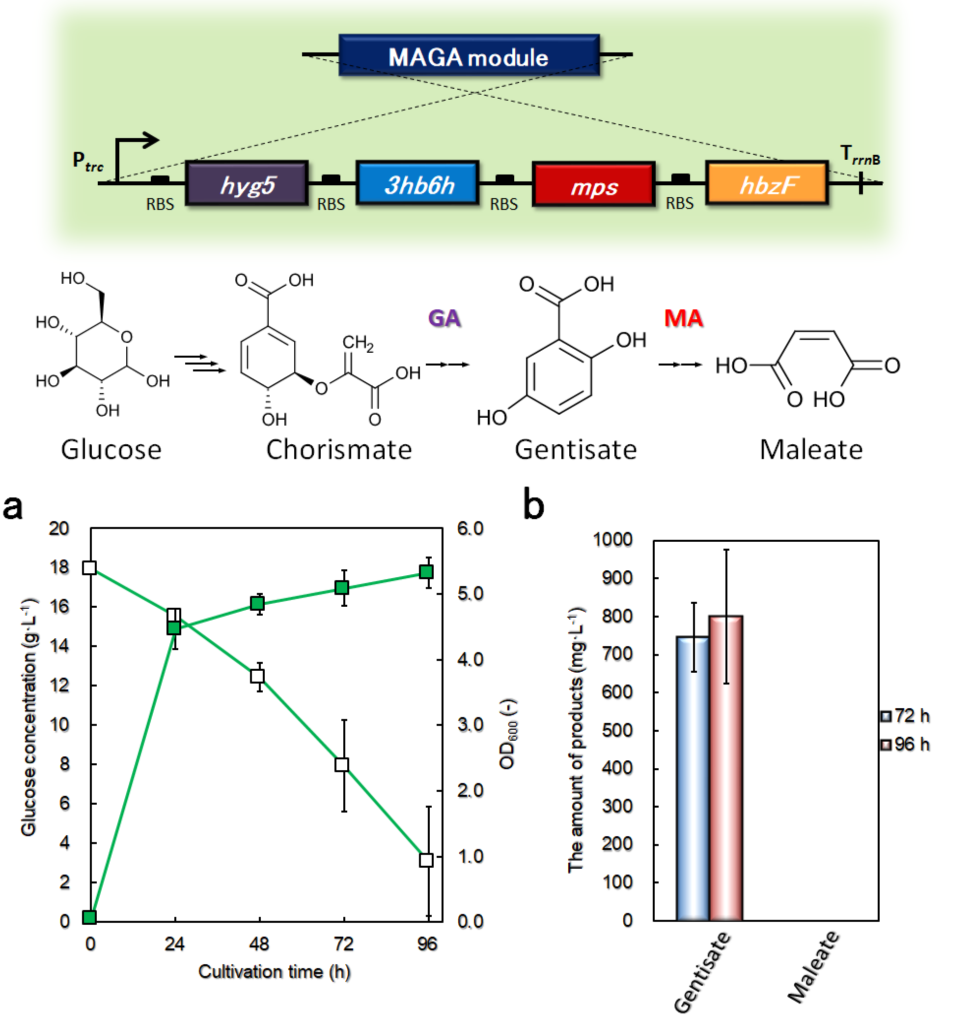

Supplementary Figure 4 | Culture profile of CFMt2 during maleate production. Time courses of (**a**) the glucose concentration (open symbols) and OD_{600} (filled symbols), and (**b**) the amounts of produced maleate and gentisate. Data are presented as the mean ± standard deviation of three independent experiments (*n* = 3).

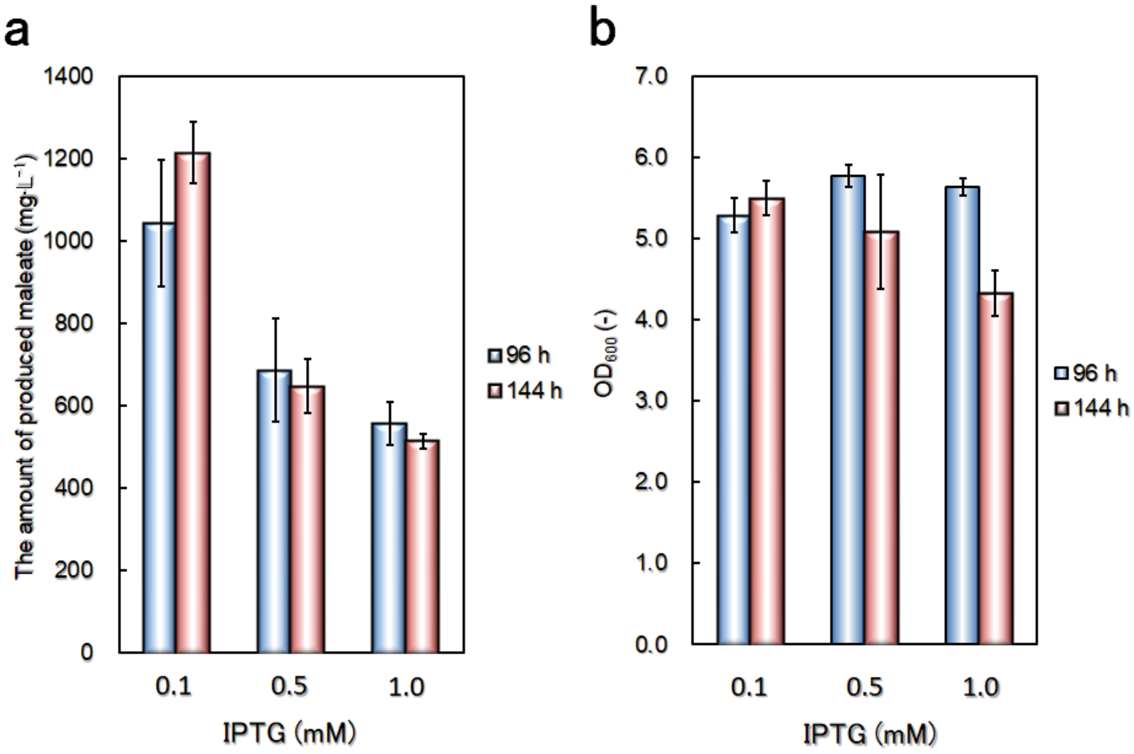

Supplementary Figure 5 | Effects of the concentration of β-D-1-thiogalactopyranoside (IPTG). (**a**) Amounts of maleate produced by CFMt1 after 96 and 144 h of cultivation. (**b**) OD_{600} values of CFMt1 after 96 and 144 h of cultivation. Data are presented as the mean ± standard deviation of three independent experiments (n = 3).

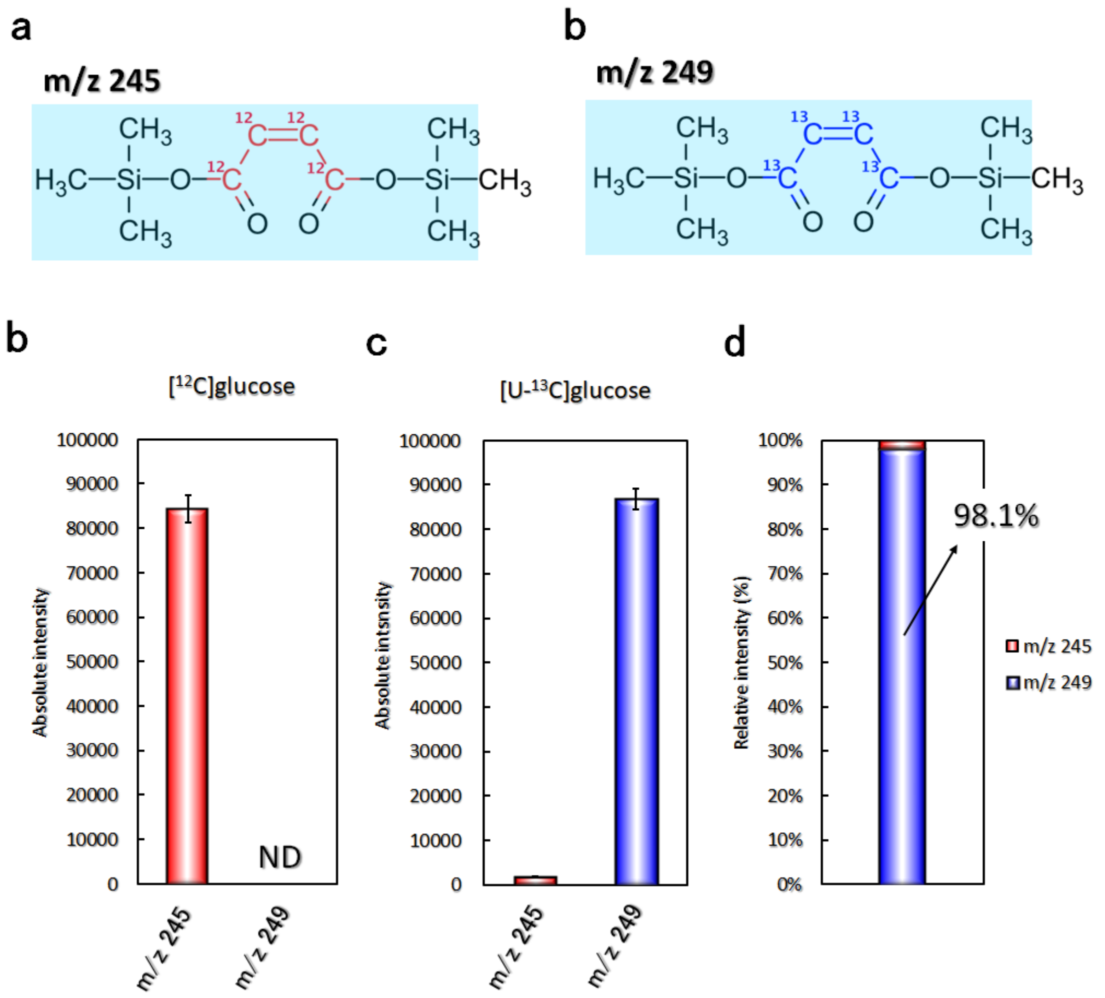

Supplementary Figure 6 | Labeling pattern of maleate produced by CMtS101 cultured using [^{12}C]glucose or [U-^{13}C]glucose as the carbon source. (**a**) Structure of maleate–bis(trimethylsilyl) ester derivative and m/z of the demethylated product. (**b**) Absolute intensity of m/z = 245 and 249 in the case of using [^{12}C]glucose. (**c**) Absolute and (**d**) relative intensity of m/z = 245 and 249 in the case of using [U-^{13}C]glucose. Data are presented as the mean ± standard deviation of three independent experiments (*n* = 3).

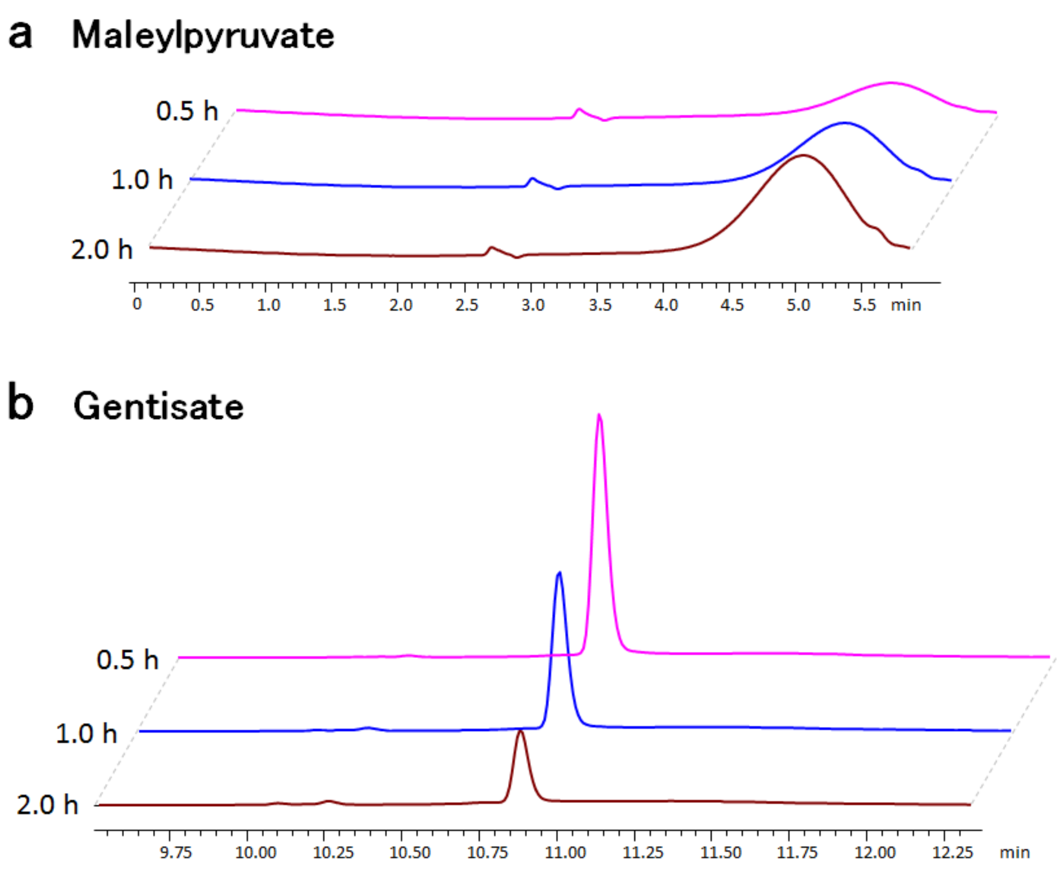

Supplementary Figure 7 | Time-course high-performance liquid chromatography chromatograms of (**a**) maleylpyruvate formation and (**b**) gentisate consumption in enzymatic conversion of gentisate to maleylpyruvate.

Supplementary Table 1. Summary of the amounts of consumed glucose and yields of produced gentisate in cultures of CFG1-3. Data are presented as the mean ± standard deviation of three independent experiments (n = 3).

	**Strains**		
	**CFG1**	**CFG2**	**CFG3**
Consumed glucose ($g \cdot L^{-1}$)	5.7 ± 0.8	5.9 ± 0.5	17.9 ± 0.6
Gentisate yield ($mol \cdot mol^{-1}$)	0.165 ± 0.022	0.130 ± 0.002	0.022 ± 0.007

Supplementary Table 2. Summary of the amounts of consumed glucose and yields of produced maleate in eight transformants. Data are presented as the mean ± standard deviation of three independent experiments (n = 3).

	Strains							
	CFM1	CFM2	CFM3	CFM4	CFM5	CFM6	CFM7	CFM8
Consumed glucose ($g \cdot L^{-1}$)	3.4 ± 0.1	4.8 ± 1.3	7.8 ± 0.6	6.0 ± 0.1	2.8 ± 0.1	1.2 ± 0.2	0.9 ± 0.2	1.1 ± 0.1
Maleate yield ($mol \cdot mol^{-1}$)	0.189 ± 0.018	0.477 ± 0.0093	0.0868 ± 0.0085	0.0842 ± 0.0086	0.117 ± 0.010	0.0896 ± 0.0401	0.0749 ± 0.0014	0.0528 ± 0.0023

Supplementary Table 3. Amounts of produced organic acids. Data are presented as the mean ± standard deviation of three independent experiments (n = 3).

Strains	Time (h)	Produced by-products ($mg{\cdot}L^{-1}$)				
		**Acetate**	**Pyruvate**	**Lactate**	**Formate**	**Malate**
CFM00	24	2,890 ± 310	21 ± 21	ND	ND	31 ± 4
	48	4,750 ± 330	76 ± 15	36 ± 3	ND	19 ± 6
	72	5,370 ± 89	157 ± 4	126 ± 10	ND	ND
CFM01	24	ND	ND	ND	ND	ND
	48	ND	ND	ND	ND	ND
	72	ND	ND	ND	ND	ND
CFM1	72	ND	ND	ND	14 ± 3	ND
	94	17 ± 30	ND	ND	17 ± 5	ND
	144	540 ± 440	ND	ND	20 ± 5	ND
CFMt1	72	ND	ND	ND	5 ± 5	ND
	94	40 ± 35	ND	ND	11 ± 3	ND
	144	640 ± 400	ND	ND	18 ± 6	ND
CFMtS09	24	ND	ND	ND	ND	ND
	48	ND	ND	ND	ND	ND
	72	82 ± 4	ND	ND	ND	ND
CFMtS10	24	ND	ND	ND	ND	ND
	48	16 ± 27	ND	ND	ND	ND
	72	74 ± 11	ND	ND	ND	ND
CFMtS091	24	ND	ND	ND	ND	ND
	48	ND	ND	ND	ND	ND
	72	ND	ND	ND	ND	ND
CFMtS101	24	ND	ND	ND	ND	ND

	48	ND	ND	ND	ND	ND
	72	ND	ND	ND	ND	ND

ND, not detected.

Supplementary Table 4. Strains, plasmids, and transformants used in this study

Strains or plasmids	Genotype	Source or reference
Strains		
NovaBlue	*endA1 hsdR17*(rK12$^-$mK12$^+$) *supE44 thi-I gyrA96 relA1 lac* recA1/F' [*proAB*$^+$ *lacI*^{q} ZΔ*M15*::Tn*10*(Tet^r)]; used for gene cloning	Novagen
BL21 (DE3) pLysS	F$^-$ *ompT hsdS*(r_B^- m_B^-) *gal dcm* λ(DE3) pLysS (Cam^r) (λ(DE3): *lac*I, *lac*UV5-T7 gene 1,*ind*1,*sam7*,*nin5*); used for protein expression	TaKaRa Bio
ATCC 31882	L-Phenylalanine-overproducing strain	American Type Culture Collection
CFT3	ATCC31882 *ptsHI*::$P_{A1lacO-1}$-*Glk-GalPΔpykFΔpykA*	[1]
CFT5	ATCC31882 *ptsHI*::$P_{A1lacO-1}$-*Glk-GalPΔpykFΔpykAΔpheΔtyrA*	[1]
CFG0	CFT5 harboring pTrcHisB	This study
CFG1	CFT5 harboring pTcgl3026	This study
CFG2	CFT5 harboring pT3hb6h	This study
CFG3	CFT5 harboring pTxlnD	This study
CFMP0	CFT5 harboring pZA23MCS	This study
CFMP1	CFT5 harboring pZA23psal	This study
CFMP2	CFT5 harboring pZA23ppu1	This study
CFMP3	CFT5 harboring pZA23palc	This study
CFMP4	CFT5 harboring pZA23rsp	This study
CFMP5	CFT5 harboring pZA23ppu2	This study
CFM1	CFT5 harboring pT2c101	This study
CFM2	CFT5 harboring pT2c102	This study
CFM3	CFT5 harboring pT2c103	This study
CFM4	CFT5 harboring pT2c104	This study

CFM5	CFT5 harboring pT2c201	This study
CFM6	CFT5 harboring pT2c202	This study
CFM7	CFT5 harboring pT2c203	This study
CFM8	CFT5 harboring pT2c204	This study
CFM9	CFT5 harboring pT2c301	This study
CFMt1	CFT5 harboring pT2t01	This study
CFMt2	CFT5 harboring pT2t02	This study
CMtS00	CFMt1 harboring pSAK	This study
CMtS01	CFMt1 harboring pS01	This study
CMtS02	CFMt1 harboring pS02	This study
CMtS03	CFMt1 harboring pS03	This study
CMtS04	CFMt1 harboring pS04	This study
CMtS05	CFMt1 harboring pS05	This study
CMtS06	CFMt1 harboring pS06	This study
CMtS07	CFMt1 harboring pS07	This study
CMtS08	CFMt1 harboring pS08	This study
CMtS09	CFMt1 harboring pS09	This study
CMtS10	CFMt1 harboring pS10	This study
CMtS091	CMtS09 harboring pA09	This study
CMtS101	CMtS10 harboring pA09	This study
BL21MP	BL21 (DE3) pLysS harboring pETM1	This study
Plasmids		
pTrcHis B	P_{trc}, pBR322 ori, Amp^r	Life Technologies
pZA23MCS	P_{AlacO1}, p15A ori, Km^r	Expressys
pZA33luc	P_{AlacO1}, luciferase gene, p15A ori, Cm^r	Expressys

pZS4Int-laci	*lacI*^{q}, SC101 ori, Spec^{r}	Expressys
pSAK	P_{AlacO1}, SC101 ori, Cm^{r}	This study
pET-22b(+)	P_{T7}, pBR322 ori, Amp^{r}	Novagen
pTcgl3026	pTrcHis B containing *hyg5* from *Streptomyces hygroscopicus* ATCC 29253 and *cgl3026* from *Corynebacterium glutamicum*	This study
pT3hb6h	pTrcHis B containing *hyg5* and *3hb6h* from *Rhodococcus jostii* RHA1	This study
pTxlnD	pTrcHis B containing *hyg5* and *xlnD* from *Pseudomonas alcaligenes* NCIMB 9867	This study
pZA23hbzF	pZA23MCS containing *hbzF* from *P. alcaligenes* NCIMB 9867	This study
pZA23psal	pZA23MCS containing *mps0* from *Pseudaminobacter salicylatoxidans* and *hbzF*	This study
pZA23ppu1	pZA23MCS containing *sgp1* from *Pseudomonas putida* and *hbzF*	This study
pZA23palc	pZA23MCS containing *mps1* from *P. alcaligenes* NCIMB 9867 and *hbzF*	This study
pZA23rsp	pZA23MCS containing *mps2* from *Rhodococcus* sp. strain NCIMB 12038 and *hbzF*	This study
pZA23ppu2	pZA23MCS containing *mps3* from *P. putida* and *hbzF*	This study
pT2c101	pT3hb6h containing *mps2* with P_{AlacO1} promoter	This study
pT2c102	pT3hb6h containing *mps3* with P_{AlacO1} promoter	This study
pT2c103	pT3hb6h containing *mps1* with P_{AlacO1} promoter	This study
pT2c104	pT3hb6h containing *sgp1* with P_{AlacO1} promoter	This study
pT2c201	pTcgl3026 containing *mps2* with P_{AlacO1} promoter	This study
pT2c202	pTcgl3026 containing *mps3* with P_{AlacO1} promoter	This study
pT2c203	pTcgl3026 containing *mps1* with P_{AlacO1} promoter	This study
pT2c204	pTcgl3026 containing *sgp1* with P_{AlacO1} promoter	This study

pTmpsR	pTrcHis B containing *mps2* and *hbzF*	This study
pT2c301	pT3hb6h containing *mps*2 and *hbzF* with P_{trc} promoter	This study
pZAt01	pZA23MCS containing *mps2*, *hbzF*, *hyg5*, and *3hb6h* in tandem	This study
pT2t01	pTrcHis B containing *mps2*, *hbzF*, *hyg5*, and *3hb6h* in tandem	This study
pT2t02	pTrcHis B containing *hyg5*, *3hb6h*, *mps2*, and *hbzF* in tandem	This study
pS01	pSAK containing $aroF^{fbr}$ with P_{AlacO1} promoter	This study
pS02	pSAK containing $aroG^{fbr}$ with P_{AlacO1} promoter	This study
pS03	pSAK containing *tktA* with P_{AlacO1} promoter	This study
pS04	pSAK containing *ppc* with P_{AlacO1} promoter	This study
pS05	pSAK containing *maeB* with P_{AlacO1} promoter	This study
pS06	pSAK containing *ppsA* with P_{AlacO1} promoter	This study
pS07	pSAK containing *glk* with P_{AlacO1} promoter	This study
pS08	pSAK containing *galP* with P_{AlacO1} promoter	This study
pS09	pSAK containing *hyg5* with P_{trc} promoter	This study
pS10	pSAK containing *hyg5* and *3hb6h* in tandem under P_{trc} promoter	This study
pA09	pZA23MCS containing *hyg5* with P_{trc} promoter	This study
pETM1	pET-22b(+) containing *mps2*	This study

Abbreviations: Amp, ampicillin; Km, kanamycin; Cm, chloramphenicol; Spec, spectinomycin.

Supplementary Table 5. Oligonucleotide primers used in this study

Oligonucleotide primers	Sequence
3hb6h_f	5′-ATCTGCAGCTGGTACTTAAAGAGGTATATAATGAGCAATCTGCAGGATGC-3′
3hb6h_r	5′-ATTCCCATATGGTACTTAGCTTGCACGATCGCTGC-3′
xlnD_f	5′-ATCTGCAGCTGGTACTTAAAGAGGTATATAATGCACAACAACATTCTGAT-3′
xlnD_r	5′-ATTCCCATATGGTACTTATTTGTATTTCTCGACGG-3′
plac_to_ptrc_f	5′-CTCCTGAGTAGGACACGAGGCCCTTTCGTCTTCAC-3′
plac_to_ptrc_r	5′-CGCTCCCGGCGGATTTCTAGGGCGGCGGATTTGTC-3′
inv_ptrc_840_f	5′-AATCCGCCGGGAGCGGATTTGAACG-3′
inv_ptrc_840_r	5′-TGTCCTACTCAGGAGAGCGTTCACC-3′
mpr_hbz_ptrc_f	5′-TATCGAATTCCTGCACGACCGGAATTATCGATTAA-3′
mpr_hbz_ptrc_r	5′-ATCCCCCGGGCTGCAATCTGTATCAGGCTGAAAAT-3′
inv_ptrc_f	5′-TATCGAATTCCTGCACGACCGGAATTATCGATTAA-3′
inv_ptrc_r	5′-ATCCCCCGGGCTGCAATCTGTATCAGGCTGAAAAT-3′
ptrc_to_ptrc_840_f	5′-CTCCTGAGTAGGACATGTTGACAATTAATCATCCG-3′
ptrc_to_ptrc_840_r	5′-CGCTCCCGGCGGATTAAGGCCCAGTCTTTCGACTG-3′
ptrc_rbs_pz_psti_f	5′-TATCGAATTCCTGCACGACCGGAATTATCGATTAA-3′
ptrc_rbs_pz_psti_r	5′-ATCCCCCGGGCTGCAATCTGTATCAGGCTGAAAAT-3′
inv_pZ_ptsi_f	5′-TGCAGCCCGGGGGATCCCAT-3′
inv_pZ_ptsi_r	5′-TGCAGGAATTCGATATCTTA-3′
mpr_to_ptrc_f	5′-CATCATCATCATGGTATGACCGCAACCGAACCGCA-3′
3hb6h_to_ptrc_r	5′-TCTCGAGCTCGGATCTTAGCTTGCACGATCGCTGC-3′
mpr_hbz_to_pt_hind_f	5′-CATATGGGAATTCGAGCGGATAACAATTTCACACA-3′
mpr_hbz_to_pt_hind_r	5′-CCGCCAAAACAGCCAACGCGTACCATGGGATCCCC-3′
cmr_f	5′-GAGCTCGATATCAAATTACG-3′

cmr_r	5′-GTGAAGACGAAAGGGCCTCG-3′
sc101_f	5′-CCTAGGGTACGGGTTTTGCT-3′
sc101_r	5′-TTTGATATCGAGCTCCGCTTGGACTCCTGTTGATA-3′
palac_f	5′-CCCTTTCGTCTTCACAAATTTATCAAAAAGAGTGT-3′
palac_r	5′-AACCCGTACCCTAGGGTCTAGGGCGGCGGATTTGT-3′
arof_f	5′-AGAGGAGAAAGGTACATGAATTATCAGAACGACGA-3′
arof_r	5′-GGGGGGGCCCGGTACTTACCCGCGACGCGCTTTTA-3′
arog_f	5′-AGAGGAGAAAGGTACATGCAAAAAGACGCGCTGAA-3′
arog_r	5′-GGGGGGGCCCGGTACTTAAGCCACGCGAGCCGTCA-3′
tkta_f	5′-AGAGGAGAAAGGTACATGTCCTCACGTAAAGAGCT-3′
tkta_r	5′-GGGGGGGCCCGGTACTTACAGCAGTTCTTTTGCTT-3′
ppc_f	5′-AGAGGAGAAAGGTACATGAACGAACAATATTCCGC-3′
ppc_r	5′-GGGGGGGCCCGGTACTTAGCCGGTATTACGCATAC-3′
maeb_f	5′-AGAGGAGAAAGGTACATGGATGACCAGTTAAAACA-3′
maeb_r	5′-GGGGGGGCCCGGTACTTACAGCGGTTGGGTTTGCG-3′
ppsa_f	5′-AGAGGAGAAAGGTACATGTCCAACAATGGCTCGTC -3′
ppsa_r	5′-GGGGGGGCCCGGTACTTATTTCTTCAGTTCAGCCA-3′
glk_f	5′-AGAGGAGAAAGGTACATGACAAAGTATGCATTAGT-3′
glk_r	5′-GGGGGGGCCCGGTACTTACAGAATGTGACCTAAGG-3′
galp_f	5′-AGAGGAGAAAGGTACATGCCTGACGCTAAAAAACA-3′
galp_r	5′-GGGGGGGCCCGGTACTTAATCGTGAGCGCCTATTT-3′
ptrc_psak_r	5′-AGTCCAAGCGGAGCTTGTTGACAATTAATCATCCG-3′
ptrc_psak_f	5′-ATTTGATATCGAGCTAAGGCCCAGTCTTTCGACTG-3′
ptrc_pza23_f	5′-TATCATGACATTAACTGTTGACAATTAATCATCCG-3′
ptrc_pza23_r	5′-AGTCAGTGAGCGAGGAAGGCCCAGTCTTTCGACTG-3′
inv_pza23_f	5′-CCTCGCTCACTGACTCGCTA-3′

inv_pza23_r	5′-GTTAATGTCATGATAATAAT-3′
mps2_pet22_f	5'-AGAAGGAGATATACAATGACCGCAACCGAACCGCA-3'
mps2_pet22_r	5'-CTCGAGTGCGGCCGCTGCACCTTCGCTAATATAGG-3'

Supplementary Table 6. Summary of proteins for maleate production.

Name	Species	NCBI accession	EC number
Hyg5	*Streptomyces hygroscopicus*	O30478.1	4.1.3.45
Cgl3026	*Corynebacterium glutamicum* ATCC 13032	Q8NLB6.1	1.14.13.24
3HB6H	*Rhodococcus jostii* RHA1	5HYM_A	1.14.13.24
XlnD	*Pseudomonas alcaligenes* NCIMB 9867	AAG39455.1	1.14.13.24
Mps0	*Pseudaminobacter salicylatoxidans*	AAQ91293	1.13.11.4
Sgp1	*Pseudomonas putida*	ACO92375.1	1.13.11.4
Mps1	*Pseudomonas alcaligenes* NCIMB 9867	AF173167_1	1.13.11.4
Mps2	*Rhodococcus* sp. strain NCIMB 12038	ADT78164.1	1.13.11.4
Mps3	*Pseudomonas putida*	WP_047604952.1	1.13.11.4
HbzF	*Pseudomonas alcaligenes* NCIMB 9867	ABD64512.1	3.7.1.23

Supplementary Note 1

Sequence of *3hb6b*:

<u>ATG</u>AGCAATCTGCAGGATGCCCGTATTATCATTGCCGGTGGTGGTATTGGTGG

TGCAGCAAATGCACTGGCACTGGCCCAGAAAGGTGCAAATGTTACCCTGTTT

GAACGTGCAAGCGAATTTGGTGAAGTTGGTGCAGGTCTGCAGGTTGGTCCG

CATGGTGCACGTATTCTGGATAGCTGGGGTGTTCTGGATGATGTTCTGAGCCG

TGCATTTCTGCCGAAAAATATCGTTTTTCGTGATGCCATTACCGCAGAAGTTC

TGACCAAAATTGATCTGGGTAGCGAATTTCGTGGTCGTTATGGTGGTCCGTAT

TTTGTTACCCATCGTAGCGATCTGCATGCAACCCTGGTTGATGCAGCACGTGC

AGCCGGTGCAGAACTGCATACCGGTGTTACCGTTACCGATGTTATTACCGAAG

GTGATAAAGCAATTGTGAGCACCGATGATGGTCGTACCCATGAAGCAGATATT

GCACTGGGTATGGATGGTCTGAAAAGCCGTCTGCGTGAAAAAATCAGCGGTG

ATGAACCGGTTAGCAGCGGTTATGCAGCATATCGTGGCACCACCCCGTATCGT

GATGTTGAACTGGATGAAGATATTGAAGATGTGGTGGGTTATATTGGTCCGCG

TTGTCATTTTATTCAGTATCCGCTGCGTGGTGGTGAAATGCTGAATCAGGTTG

CAGTTTTTGAAAGTCCGGGATTCAAAAACGGCATTGAAAATTGGGGTGGTCC

GGAAGAACTGGAACAGGCATATGCACATTGCCATGAAAATGTTCGTCGCGGT

ATTGATTATCTGTGGAAAGATCGTTGGTGGCCGATGTATGATCGTGAACCGAT

TGAAAACTGGGTTGATGGTCGCATGATTCTGCTGGGTGATGCCGCACATCCG

CCTCTGCAGTATCTGGCAAGCGGTGCAGTTATGGCAATCGAAGATGCAAAAT

GTCTGGCAGATTATGCAGCCGAAGATTTTAGCACCGGTGGTAATAGCGCATGG

CCTCAGATTCTGAAAGAAGTTAATACCGAACGCGCACCGCGTTGTAATCGTAT

TCTGACCACCGGTCGTATGTGGGGTGAACTGTGGCATCTGGATGGCACCGCA

CGTATTGCACGTAATGAACTGTTTCGTACCCGTGATACCAGCAGCTATAAATA

CACCGATTGGCTGTGGGGTTATAGCAGCGATCGTGCAAGCTAA

Sequence of *xlnD*:

ATGCACAACAACATTCTGATTGCCGGTGCAGGTATTGGTGGTCTGAGCGCAG

CACTGGGTCTGGCACGTAAAGGTATGCGTAGCATTGTTCTGGAAAAAGCACC

GGAACTGGGTGAAATTGGTGCCGGTATTCAGCTGGCACCGAATGCATATCAT

GCACTGGATGCCCTGGGTATTGGCGAAGTTGCACGTCAGACCGGTGTTCATG

TTGATAAACTGCTGTGGATGGATGGTATGACCGATAAAGAAATTGCAAGCGTT

CCGCTGGCAAATCGTTTTCGTGAATTTTTTGGTAATCCGTATGCCGTTATTCAC

CGTGCAGATTTTCATGGTCTGCTGGTTGAAGCATGTCATAAAACCGGTCTGGT

GGAAGTTCGTACCAATGCCGAAGTTGTTGATTATGAAAACTTTCCGGATCGTG

TTGAAGCCATTCTGCATGATGGTAGCTGTATTAATGGTGCAGTTCTGGTTGGT

GCAGATGGTCTGTGGTCAAATGTTCGTCAGAAAGTTATTGGTGATGGTGATCC

GCGTGTTAGCGGTCATACAACCTATCGTAGCGTTATTCCGGCAGAAGATATGC

CGGAAGAACTGCGTTGGAATATGAGCACCGCATGGGCAGGCGAAGGTTGTC

ATATGGTTCATTATCCGCTGAAAGGTGGCAAAGTTTTAATCTGGTTCTGACC

AGCAATAGCGGTGCAAGCGAACCGGAAGCCGGTGTTCCGGTTACCACCGAT

GAAGTTTTTGAAAAATTCAAAACCATGAAACGTCGTCCGACCAGCCTGATTC

ATAAAGGTAATAATTGGAAACGTTGGGTGCTGTGCGATCGTGATCCGCTGCCG

AATTGGGTTGATGGTCGTGTTACCCTGCTGGGTGATGCAGCACATCCGATGAT

GCAGTATATGGCACAGGGTGCAAGCATGGCAATTGAAGATGCAGTTTGTCTG

GCATTTGAACTGGGTCGTGAAATGGATCCGGTTAGCGCACTGAAAAAATACA

ATCGTGCACGTTTTGCACGTACCGCACGTGTTCAGACCTATAGCCGTTATGCA

AGCGATTTTATCTATCATGCAAAAGGTGGTGCAGCAGCAATGCGTAATGAACT

GATGGGTGGCATGACCCCGACCGATTTTTTCCAGTGGATTAATTGGCTGTATG

GCAAAGAAACCGTCGAGAAATACAAATAA

Sequence of *cgl3026*:

ATCTGCAGCTGGTACTTAAAGAGGTATATAATGAGCCTGCCGCATAGTGATGA

ACTGCGTGGTCAGAAAATTATCATTAGCGGTGGTGGTATTGGTGGTGCAGCC

GGTGCACTGGCACTGGCCCTGCGTGGTGCCGATGTTACCCTGTATGAACGTG

CAGCAGAATTTAAAGAAGTTGGCGCAGGTCTGCAGATTGGTCCGCATGGTTG

GCGTATGCTGGAAAGCTGGGGTCTGCTGGATCAGATTGTTGTTGCAGGTTATC

TGCCGGAAGATATGCAGTTTCGTGATGCAGTTAATCGTGAAACCATTCTGACC

ATGCGTTTTGATGAAGAATTTCAGCAGCATTATGGTGGTCGTTATCTGGTTATT

CATCGTAGCGATCTGCTGAATATCCTGGTTACCAATGCCGAAGCAGCGGGTGC

AAAACTGCACAATGGTGTTCTGGTTACCGATAGCCGTACCGTTGATGGTGGCA

TTGAAGTTGATATTGAAAGCAGCATCAATAAAGGCGAGGATAACAAAACCCT

GCTGGTTGATGCATTTCTGGCATTTGATGGTATTCATAGCGTGATGCGTAAAA

AACTGGTGGATGATGCACCGGTTGCAAGCAGCTATGTTGCATATCGTGGCAC

CAGCAAACTGGCCGAAGATGCAGAAATGAAAGATCTGAAAAGCGTGATTGG

TTATATTGGACCGCATGTGCATTTTATTCAGTATCCGCTGCGTGGCGGTGAACT

GCTGAATCAGGTTGCAGTTTTTGAAAGCCAGCGCTATCTGGATGGTCGTACC

GCAGGCGATATTCCGGAAGATTGGGGTAATCCGGAAGAACTGGATCGTGCAT

ATAATCATTGTGATCCGTTTATTCAGGATCGTCTGGATACCCTGTGGCGTAATA

ATTGGTGGCAGATGAGCGATCGTGAACCGCTGGAAAATTGGCGTATTGGTCG

CATGCTGCTGCTGGGAGATGCAGCACATGCACCGCTGCAGTATCTGGCAAGC

GGTGCAGTTATGGCAATGGAAGATGCGGAAGCAGTTGCACTGTTTGCAGCAG

ATGCAGCCCGTGCAGGTAATCTGGATTGGGAAGAAGTTCTGGCAGAAGTTGA

AGCAGAACGTCGTCCGCGTTGTAGCCGTATTCAGACCGTTGGTCGTTTTTGG

GGTGAACTGTGGCATGTTGAAGGCACCGCACGTCTGATTCGTAATGAAGTTT

TTCGTCAGGCAGATCGTAATGGCTGGTTTATCTATGCCGATTGGCTGTGGGGT

TATGATGCAAGCAAACGTGCACATATTGCAAATCCGGAACTGGGTGAAATGC

CGCAGGCACTGAAAGAATGGCGTTATGCACTGCTGGAACAGAAATAAGTAC

CATATGGGAAT

Sequence of *hbzF*:

GTCGACGGTATCGATTTCATTAAAGAGGAGAAAGGTATGAAAATCTGCCGCT

TTAATGAAAATCGTCTGGGTGTTGTTGATGGTGATGAAGTTCTGGATGTTACC

GAAGCACTGAGCGTTCTGCCGAGCTATGAATATCCGCTGCCTGGTTATGATCC

GCTGATTAAACATCTGGATGCACTGCTGGCACGTATTGAAACCCTGATTAAAG

ATGCACCGCGTATTCTGCTGGCAGATGTTCGTCTGCTGAGTCCGGTTGCAAAT

CCGGGTAAAATCATTGCAGCACCGATTAACTATACCCGTCATCTGCAAGAAGT

GCTGGCCGATAGCGCAATTAACAATGGTGTTGCAAGCTTTACCCAGCATATCA

AAAAAAGCGGTCTGTTTCTGAAAGCAAATAGCAGCCTGGCTGGTGCCGGTG

AAGGTGTTGCACTGAGCCATCAGGATCGTCGTAATGATCATGAAGTTGAACT

GGCCATTGTGATTGGTAAAACCGCACGTAATGTTCCGCGTGAAAAAAGCCTG

GAATATGTTGCAGGTTATTGCATTGGTATTGATATGACCGTTCGTGGTCCGGAA

GAACGTAGCTTTCGTAAAAGTCCGGATAGCTATACCATTCTGGGTCCGTGGCT

GGTTACCCGTGATGAAATTGATAGTCCGGGTGAACTGCAGATGAGCCTGAAA

GTTAATGGTGAAGTTCGTCAGAATGCAAATACCAGCGATCTGATTCTGGGTGT

GGAAGAACTGGTTGAATTTGCAAGCAGCTTTTATACCCTGCATCCGGGTGAT

GTGATTATTAGCGGTACACCGGAAGGTGTGGGTCCGGTTAATCCTGGTGATGC

CATGCTGGCGGAAATTGAACGTATTGGCACCATGACCATTGCAATTCGTAGCG

TTGACTACAAGGATGACGATGACAAG<u>TAA</u>GATATCGAATTCCTG

Sequence of *mps0*:

ATTAAAGAGGAGAAAGGTACC<u>ATG</u>CAGAACGAAAAACTGGATCATGAAAGC

GTTACCCAGGCAATGCAGCCGAAAGATACACCGGAACTGCGTGCACTGTATA

AAAGCTTTGAAGAAGAAAGCATTATTCCGCTGTGGACACAGCTGGGTGATCT

GATGCCGATTCATCCGAAAAGCAAAGCAGTTCCGCATGTTTGGAAATGGTCA

ACCCTGCTGCGTCTGGCACGTAAAAGCGGTGAACTGGTTCCGGTTGGTCGTG

GTGGTGAACGTCGTGCACTGGGTCTGGCAAATCCGGGTCTGGGTGGTAATGC

ATATATTAGCCCGACCATGTGGGCAGGTATTCAGTATCTGGGTCCGCGTGAAA

CCGCACCGGAACATCGTCATAGCCAGAATGCATTTCGTTTTGTTGTTGAAGGT

GAAGGTGTTTGGACCGTTGTTAATGGTGATCCGGTTCGTATGAGCCGTGGTGA

TCTGCTGCTGACACCGGGTTGGTGTTTTCATGGTCATCATAATGATACCGATC

AGCCGATGGCATGGATTGATGGTCTGGATATTCCGTTTAGCCAGCAGATGGAT

GTTGGCTTTTTTGAATTTGGTAGCGATCGTGTTACCGATTATGCAACCCCGAAT

TTTAGCCGTGGCGAACGTCTGTGGTGTCATCCTGGTCTGCGTCCGCTGAGCG

GTCTGCAGAATACCGTTGCAAGCCCGATTGGTGCATATCGTTGGGAATTTACC

GATCGTGCCCTGACCGAACAGCTGCTGCTGGAAGATGAAGGTCAGCCTGCA

ACCGTTGCACCGGGTCATGCAGCAATTCGTTATGTTAATCCGACCACAGGTG

GTGATGTTATGCCGACCCTGCGTTGTGAATTTCATCGTCTGCGTGCAGGCACC

GAAACCGCAACCCGTAATGAAGTTGGTAGCACCGTTTTTCAGGTTTTTGAAG

GTGCCGGTGCAGTTGTTATGAATGGTGAAACCACCAAACTGGAAAAAGGCG

ATATGTTTGTTGTTCCGAGCTGGGTTCCGTGGTCACTGCAGGCAGAAACCCA

GTTTGACCTGTTTCGTTTTAGTGATGCTCCGATTATGGAAGCACTGAGCTTTAT

GCGTACCAAAATTGAAGGCCAGAAACATCATCATCATCATCATTAAGGGCCCC

CCCTCGAG

Sequence of *sgp1*:

ATTAAAGAGGAGAAAGGTACCATGAATACCGCACCGAATACCCAGAGCACCA

GCGCACGTGCAGCATATTATCGTCGTATTGATGCATTTGCACTGACACCGCTG

TGGGAAAGCCTGCATGCACTGGTTCCGAAAACCCCGACCACCGCAATTGAA

CCGGCACTGTGGCGTTATGCAGAAGTTCGTCCGCTGCTGGCAGAAAGCGGTG

AACTGATTAGCGCAATGGAAGCAGAACGTCGTGTTCTGATTCTGGAAAATCC

GGCACTGCGTGGTCAGAGCGCAATTACCCAGAGTCTGTATGCAGGTCTGCAG

CTGATTCTGCCTGGTGAAGTTGCACCGGCACATCGTCATAGCCAGAGCGCAC

TGCGTCTGGTTCTGGAAGGTGAAACCGCATTTACCGCAGTTGATGGTGAACG

TATTCCGATGACACCGGGTGATTTTATCATTACTCCGGCACATACCTGGCATGA

TCACGGTAATACCGGTAATGATGCAGTTGTTTGGCTGGATGGTCTGGATATTC

CGCTGGTTCGTTTTTTTGAAGCAGGTTTTGCAGAAAAAGCCGAAGAAAGCG

TTCAGCGTGTTAGCCGTGCAGAAGGTGATAGCCTGGCACGTTATGGTAGCAA

TCTGGTTCCGCTGGATAGCCGTCGTGATGCAGCACAGCCGAGCCGTCTGTGT

CATTATCCGTATAAACATACCCGTGCAGCACTGGCAGATCTGGCACGTGGTGA

TGCAGATCCGCATTATGGTCATGCACTGCGTTTTAACAATCCGGAAACCGGTC

ATAGCCCGATGCCGACCATTGGCACCTTTATTCAGCTGCTGCCTGCCGGTTTT

GAAACCCGTACCCGTCGTAGCACCGATAGCACCATTGTTTGTTGTCTGGAAG

GCGGTGGCACCGCACAGGTTGGTGAACGCACCTTTCAGTTTGCACAGAATGA

TATTTTTGTGATCCCGAGCTGGTCAGCCCTGCGTCTGCAGGCAGGTCCGGAA

ACAGTTCTGTTTAGCTATAGCGATCGTCCGGTTCAGCAGGCACTGGGTCTGTG

GAAAGAAGAACGTATTCATCATCATCATCATCATTAAGGGCCCCCCCTCGAG

Sequence of *mps1*:

ATTAAAGAGGAGAAAGGTACCATGAGCTTTACCGAAAAACCGGCAGTTACC

AAAGAACGCAAAGAGTTTTATAGCAAACTGGAAAGCCATGATCTGGCACCG

CTGTGGGAAGTTCTGAATGAAGTTGTTACCACCAAACCGAAAAGCAATTGTG

CACCGCATCTGTGGGAATTTGAAGTTGCAAAAGAATTTCTGATGGAAGCAGG

CACCCTGATTACCGCAAAAGAAGCAGAACGTCGCGTACTGATTCTGGAAAAT

CCGGGTCTGAAAGGTCTGAGCCGTATTACCACCAGTCTGTATGCAGGTCTGC

AGCTGATTCTGCCTGGTGAAGTGGCACCGACCCATCGTCATAGCCAGAGCGC

ACTGCGTTTTGTTGTTGATGGTGGTGGCGCATGTACCAGCGTGGATGGTGAA

CGTACCACCATGCAGGTTGGTGATTTTGTTATTACCCCTCCGTGGGCATGGCA

TGATCACGTTAATGATAGCGATAAACCGATGATTTGGATGGATGGTCTGGATC

TGCCGATGGTTACCCTGTTTGATACCAGCTTTGCCGAAGGTTATGGTGAAGAT

ATTCAAGAAATTACCCGTCCGAATGGTGATAGCCTGGCACGTTATGGTGCAAA

TATGCTGCCGGTTGATTTTAAACAGAAAGGCCTGAGCAGCCCGATTTTTAACT

ATCCGTATGAACGTAGCCGTGAAGCACTGGAAGCAATGAAAAAAGCAAATG

AATGGGATCCGTGTCATGGTCTGAAAATGCAGTATATTAACCCGCTGGATGGT

ATGGCAGCAATGCCGACCATTAGCAGCTTTATTCAGCTGCTGCCGAAAGAATT

CCGTACCCAGACCTATCGTAGCACCGATGCAACCGTTTTTAGCGTTATTGAAG

GTCAGGGTAAAACCCGCATTGGTGATAAAGTGTTTTTCTGGAAAGCGAAAGA

TACCTTTGTTGTTCCGAGTTGGTATCCGGTTGAACATGAAGCAAGCAGTGATG

CAGTTCTGTTTAGCTATAGCGATCGTGTTGCACAGCAGAAACTGGGTTTTTGG

CGTGAAAGCCGTAATCATCATCATCATCATCATTAAGGGCCCCCCCTCGAG

Sequence of *mps2*:

ATTAAAGAGGAGAAAGGTACCATGACCGCAACCGAACCGCATGTTGATGATG

ATCCGGCACTGAAACAGCTGTATACCGATTTTGAAGCAGAACATCTGAATCC

GCTGTGGACACAGCTGGGTGATCTGATGCCGATGACCCCGACCAGCCGTGCA

GTTCCGTTTGTTTGGAAATGGTCAACCCTGTATCCGCTGGCACAGCGTGCCG

GTGATCTGGTTCCGGTTGGTCGTGGTGGTGAACGTCGTGCAATTGCCCTGGC

AAATCCGGGTCTGGGTGGTGTTCCGTATGTTACCCCGACCCTGTGGGCAGCA

ATTCAGTATCTGGGTCCGAAAGAAGTTGCACCGGAACATCGTCATGCACAGA

ATGCATTTCGTTTTGTTGTTGAAGGTGAAGGTGTTTGGACCGTTGTTAATGGT

GATCCGGTTGCAATGCGTCGTGGTGATTTTCTGCTGACACCGGGTTGGCATTT

TCATGGTCATCATAATGAAACCGATCAGCCGATGGCATGGATTGATGGTCTGG

ATATCCCGTTTGTTCATTATACCGATACCGGCTTTTTTGAATTTGGCAGCGAAA

ATGTTACCGATGATAGCACACCGGATGTTAGCCGTAGCGAACGTCTGTGGGC

ACATCCTGGTCTGCGTCCGCTGGTTGGCCTGGATGCAAAAACCAGCAGCCCG

ATTGCAGCATATCGTTGGGAACACACCGATGCAGCACTGCGTGAACAGCTGG

CACTGGAAGATGAAGGTTATGCAGCAACCACCGAACCGGGTCATGCAGCAG

TTCGTTATACCAATCCGACCACAGGTGGTGATGTTATGCCGACCATTCGTGCA

GAATTTCATCGTCTGCGTGCGGGTGCACATACCCGTCCGCGTCGTGATGTTGG

TAGCACCGTTTATCAGGTTTTTGAAGGTAGCGGTCGTTTTGTGCTGGGTGGTC

AGACCCGTACCGTGGGTAAAGGTGATATGATTGTTGTTCCGAGCTGGACCGA

ATGGTCAATTGAAGCCGATACCGAATTTGACCTGTTTGCATTTAGTGATGCAC

CGATTGTTGAACGTCTGCATTTTCACCGTACCTATATTAGCGAAGGTGCACAT

CATCATCATCATCATTAAGGGCCCCCCCTCGAG

Sequence of *mps3*:

ATTAAAGAGGAGAAAGGTACCATGAACAACAACGATAATATTGGCACCGCAC

GTGCAGATTTTTATCAGCGTATTGATAGCCAGGCACTGTTTCCGCTGTGGGAA

CAGCTGCATAATCTGGTTCCGCCTCAGCCGACCACCACCTGTGTTCCGGCAC

TGTGGCGTTTTAGCGAAGTTCGTCCGTATCTGATGGAAGCAGGTCAGCTGATT

AGTGCCGAAGAAGCAGTTCGTCGCGTTCTGGTTCTGGAAAATCCGGGTATTC

GTGGTCAGGCAAGCATTACCCCGAGCCTGTATGCAGGTCTGCAGCTGATTCT

GCCTGGTGAAATTGCACCGAGCCATCGTCATAGCCAGAGCGCACTGCGTTTT

GTTGTTGAAGGTAAAGGTGCATATACCGCAGTTGATGGTGAACGTACCACCA

TGCATCCGGGTGATTTCATTATTACCCCGTCATGGACCTGGCATGATCACGGTA

ATCCGAGCGAAACCGAAGGTGGTGAACCGGTTATTTGGCTGGATGGTCTGGA

TATTCCGACCGTTCGTTTTTATGGTGCAGGTTTTGCAGAAAATTATCCGCAGG

CAGTTCAGCCGGTTTCACGTCCGGATGGTAATAGCCTGGCACGTTATGGTGCG

AATATGCTGCCGCTGCGTCATGAAGTTCAGGGTGCAACCAGCCCGATTTTTAG

CTATCCGTATGCACGTACCCGTGAAGCACTGGCAACCCTGGAACAGCAGGGT

GAAGTTGATGCATGGGATGGTGTTAAACTGCGTTATGTTAATCCGGCAACCGG

TGGTTGGGCAATGCCGACCATTGGTACATGCATGCAGCTGCTGCCTCGTGGTT

TTGCGGGTAAAACCGCACGTTGTACCGATGCAACCGTTTATAGCGTTGTGGA

AGGTCGTGGTCGTGCAATTATTGGTGGTCAGACCTTTCATTTTGAACCGAAA

GATACCTTTGTTGTTCCGAGCTGGGTGACCCTGAGTCTGCAGGCAGATGATG

CATGTGTTCTGTTTAGCTATAGCGATCGTCCGGTTCAAGAAGCCCTGGGTCTG

CTGCGTGAAGCCCGTGAACATCATCATCATCATCATTAAGGGCCCCCCCTCGA

G

Sequence of *aroF*^{fbr}:

GGATCCATTAAAGAGGAGAAAGGTACCATGCAAAAAGACGCGCTGAATAAC

GTACATATTACCGACGAACAGGTTTTAATGACTCCGGAACAACTGAAGGCCG

CTTTTCCATTGAGCCTGCAACAAGAAGCCCAGATTGCTGACTCGCGTAAAAG

CATTTCAGATATTATCGCCGGGCGCGATCCTCGTCTGCTGGTAGTATGTGGTCC

TTGTTCCATTCATGATCCGGAAACTGCTCTGGAATATGCTCGTCGATTTAAAG

CCCTTGCCGCAGAGGTCAGCGATAGCCTCTATCTGGTAATGCGCGTCTATTTT

GAAAAACCCCGTACCACTGTCGGCTGGAAAGGGTTAATTAACGATCCCCATA

TGGATGGCTCTTTTGATGTAGAAGCCGGGCTGCAGATCGCGCGTAAATTGCT

GCTTGAGCTGGTGAATATGGGACTGCCACTGGCGACGGAAGCGTTAGATctgA

ATAGCCCGCAATACCTGGGCGATCTGTTTAGCTGGTCAGCAATTGGTGCTCGT

ACAACGGAATCGCAAACTCACCGTGAAATGGCCTCCGGGCTTTCCATGCCGG

TTGGTTTTAAAAACGGCACCGACGGCAGTCTGGCAACAGCAATTAACGCTAT

GCGCGCCGCCGCCCAGCCGCACCGTTTTGTTGGCATTAACCAGGCAGGGCAG

GTTGCGTTGCTACAAACTCAGGGGAATCCGGACGGCCATGTGATCCTGCGCG

GTGGTAAAGCGCCGAACTATAGCCCTGCGGATGTTGCGCAATGTGAAAAAGA

GATGGAACAGGCGGGACTGCGCCCGTCTCTGATGGTAGATTGCAGCCACGGT

AATTCCAATAAAGATTATCGCCGTCAGCCTGCGGTGGCAGAATCCGTGGTTGC

TCAAATCAAAGATGGCAATCGCTCAATTATTGGTCTGATGATCGAAAGTAATA

TCCACGAGGGCAATCAGTCTTCCGAGCAACCGCGCAGTGAAATGAAATACG

GTGTATCCGTAACCGATGCCTGCATTAGCTGGGAAATGACCGATGCCTTGCTG

CGTGAAATTCATCAGGATCTGAACGGGCAGCTGACGGCTCGCGTGGCTTAAT

CTAGAGGCATCAAATAAAACAAGCTT

Sequence of $aroG^{fbr}$:

GGATCCATTAAAGAGGAGAAAGGTACCATGAATTATCAGAACGACGATTTAC

GCATCAAAGAAATCAAAGAGTTACTTCCTCCTGTCGCATTGCTGGAAAAATT

CCCCGCTACTGAAAATGCCGCGAATACGGTTGCCCATGCCCGAAAAGCGATC

CATAAGATCCTGAAAGGTAATGATGATCGCCTGTTGGTTGTGATTGGCCCATG

CTCAATTCATGATCCTGTCGCGGCAAAAGAGTATGCCACTCGCTTGCTGGCGC

TGCGTGAAGAGCTGAAAGATGAGCTGGAAATCGTAATGCGCGTCTATTTTGA

AAAGCCGCGTACCACGGTGGGCTGGAAAGGGCTGATTAACGATCCGCATATG

GATAATAGCTTCCAGATCAACGACGGTCTGCGTATAGCCCGTAAATTGCTGCT

TGATATTAACGACAGCGGTCTGCCAGCGGCAGGTGAGTTTCTCaacATGATCA

CCCCACAATATCTCGCTGACCTGATGAGCTGGGGCGCAATTGGCGCACGTAC

CACCGAATCGCAGGTGCACCGCGAACTGGCATCAGGGCTTTCTTGTCCGGTC

GGCTTCAAAAATGGCACCGACGGTACGATTAAAGTGGCTATCGATGCCATTAA

TGCCGCCGGTGCGCCGCACTGCTTCCTGTCCGTAACGAAATGGGGGCATTCG

GCGATTGTGAATACCAGCGGTAACGGCGATTGCCATATCATTCTGCGCGGCGG

TAAAGAGCCTAACTACAGCGCGAAGCACGTTGCTGAAGTGAAAGAAGGGCT

GAACAAAGCAGGCCTGCCAGCACAGGTGATGATCGATTTCAGCCATGCTAAC

TCGTCCAAACAATTCAAAAAGCAGATGGATGTTTGTGCTGACGTTTGCCAGC

AGATTGCCGGTGGCGAAAAGGCCATTATTGGCGTGATGGTGGAAAGCCATCT

GGTGGAAGGCAATCAGAGCCTCGAGAGCGGGGAGCCGCTGGCCTACGGTAA

GAGCATCACCGATGCCTGCATCGGCTGGGAAGATACCGATGCTCTGTTACGTC
AACTGGCGAATGCAGTAAAAGCGCGTCGCGGG<u>TAA</u>TCTAGAGGCATCAAATA
AAACAAGCTT

Underline: Initiation and termination codons

*The codons encoding amino acid changes with respect to the original sequence are indicated as small letters.

Supplementary references

1. Noda, S., Shirai, T., Oyama, S. & Kondo, A. Metabolic design of a platform *Escherichia coli* strain producing various chorismate derivatives. *Metab. Eng.* **33**, 119–129 (2016).
